# ROD1 Is a Seedless Target Gene of Hypoxia-Induced miR-210

**DOI:** 10.1371/journal.pone.0044651

**Published:** 2012-09-14

**Authors:** Pasquale Fasanaro, Sveva Romani, Christine Voellenkle, Biagina Maimone, Maurizio C. Capogrossi, Fabio Martelli

**Affiliations:** 1 Istituto Dermopatico dell’Immacolata IRCCS, Roma, Italy; 2 IRCCS Casa Sollievo della Sofferenza, San Giovanni Rotondo, Italy; 3 IRCCS Policlinico San Donato, Milano, Italy; Northwestern University, United States of America

## Abstract

Most metazoan microRNA (miRNA) target sites have perfect pairing to the “seed” sequence, a highly conserved region centering on miRNA nucleotides 2–7. Thus, complementarity to this region is a necessary requirement for target prediction algorithms. However, also non-canonical miRNA binding can confer target regulation. Here, we identified a seedless target of miR-210, a master miRNA of the hypoxic response. We analyzed 20 genes that were inversely correlated to miR-210 expression and did not display any complementarity with miR-210 seed sequence. We validated ROD1 (Regulator of Differentiation 1, also named PTBP3, Polypyrimidine Tract Binding protein 3) as a miR-210 seedless transcript enriched in miR-210-containing RNA-induced silencing complexes. ROD1 was not indirectly targeted by a miR-210-induced miRNA. Conversely, we identified a “centered” miR-210 binding site in ROD1 involving 10 consecutive bases in the central portion of miR-210. Reporter assays showed that miR-210 inhibited ROD1 by the direct binding to this sequence, demonstrating that ROD1 is a *bona fide* seedless target of miR-210. As expected, both ROD1 mRNA and protein were down-modulated upon hypoxia in a miR-210 dependent manner. ROD1 targeting by miR-210 was biologically significant: the rescue of ROD1 inhibition significantly increased hypoxia-induced cell death. These data highlight the importance of ROD1 regulation by miR-210 for cell homeostasis.

## Introduction

The rules that guide miRNA/mRNA interactions are very complex and still under intense investigation [1]. The current paradigm states that a Watson–Crick pairing between the 3′UTR region of the target mRNA and the 5′ region of the miRNA centered on nucleotides 2–7, termed “seed sequence”, is required for miRNA-mediated inhibition [1]. Thus, the seed pairing is a necessary requirement for target prediction algorithms. In the last few years, experimental approaches aimed to the unbiased identification of miRNA targets have been undertaken by several groups [2]. The results revealed that also non-canonical miRNA binding can confer target regulation [3]. It has been demonstrated that certain mRNAs are targeted by miRNAs recognizing their coding sequence (*cds*) or their 5′UTR [4]. Moreover, in 2009 Lal et al. described for the first time a “seedless” miRNA/mRNA interaction, demonstrating that miR-24 inhibits cell proliferation by targeting E2F2, MYC, and other cell-cycle genes by a seedless pairing [5]. Other miRNA binding patterns, such as seed sites with G:U-wobbles and G-bulges within the seed, 3′ compensatory sites and seedless centered sites, were recently described [3], [6], [7], confirming that the complexity of miRNA activity is far from being elucidated.

miR-210 can be considered a master miRNA of the hypoxic response and is currently regarded as a promising novel non-invasive tumor hypoxia marker [8]–[10]. The instrumental role of miR-210 in the regulation of cell response to hypoxia is confirmed by numerous pre-clinical and clinical evidences. Indeed, miR-210 has been found to be up-regulated upon brain transient focal ischemia in rats and after human myocardial infarction [11], [12]. Moreover, circulating miR-210 was proposed as biomarker in acute cerebral ischemia [11] and acute kidney injury [13]. The targets identified to date indicate that miR-210 plays a role in cell cycle regulation, differentiation, mitochondrial metabolism repression, DNA repair and apoptosis [8], [14]–[17].

Specifically, our group demonstrated that miR-210 up-regulation plays an integral role in endothelial cell adaptation to hypoxia [18]. We found that miR-210 increases endothelial tubulogenesis and migration, whereas miR-210 blockade in the presence of hypoxia inhibits these processes and induces apoptosis. We also applied an integrated strategy for large-scale identification of miR-210 targets [15]. We selected candidate miR-210 targets by a combination of bioinformatics, proteomics and transcriptomics. These candidates were then screened for the presence of miR-210-seed complementary sequences and validated for their enrichment in miR-210-containing RNA-induced silencing complexes (RISC). RISC-immunoprecipitation is currently accepted as a state of the art technique for the identification of genuine miRNA targets [2]. However, while this approach allowed the identification of 32 new miR-210 targets, it did not keep in account possible non-canonical miRNA/target interactions.

In this follow-up study, we assayed 20 genes displaying a reciprocal modulation with miR-210, but excluded by our previous validation because seedless. We identified ROD1 (Regulator of Differentiation 1, also named PTBP3, Polypyrimidine Tract Binding protein 3) as a *bona fide* seedless miR-210 target, likely involved in the anti-apoptotic activity of miR-210.

## Materials and Methods

### Cell Cultures

HEK-293 cells (ATCC) were grown in Dulbecco’s modified Eagle’s medium (DMEM) containing 10% FBS. Commercially available human umbilical vein endothelial cells (HUVEC; Clonetics/Lonza) were grown in EGM-2 (Lonza) containing 2% FBS. Hypoxic conditions were maintained in defined atmosphere chambers (Billups-Rothenberg Inc.); cells were placed in the chamber and then a gas mixture of 5% CO_2_ - 95% N_2_ was injected for 20 min, yielding about 1% O_2_ [18]; thereafter, the chamber was sealed and incubated at 37°C for the indicated time. ROD1 over-expression was obtained by transfection of pCMV6-XL5-ROD1 (clone number NM_005156.3, OriGene) using Fugene6 (Roche). Cell death was assessed by Trypan blue exclusion. Lentiviral infections were performed as previously described [19].

### Immuno-precipitation of c-myc-Ago2-containing RISC

Transfected cells were harvested in 1 ml/p15 dish of cold lysis buffer (150 mM KCl, 25 mM Tris-HCl pH 7.4, 5 mM EDTA, 0.5% NP40) supplemented with 5 mM dithiothreitol, 1 mM phenylmethylsulfonyl fluoride, protease inhibitors mixture tablets (Roche) and 100 u/ml of RNasin Plus (Promega). After 30 min at 4°C, samples were pre-cleared for 10 min with 75 µl/plate of A/G-agarose beads (Santa Cruz) and spun at 4°C for 30 minutes at 20.000 *g* in a micro-centrifuge. Next, lysates were incubated at 4°C with 2.5 µg/plate of anti-c-myc antibody (9E10, Santa Cruz) for 3 hrs and then 50 µl/plate of A/G-agarose beads were added to each sample. After 1 hr, immuno-complexes were washed two times with lysis buffer and resuspended in 200 µl of TRIzol (Invitrogen). RNA was purified and specific mRNAs were measured by quantitative real-time PCR (qPCR). Average values of 3 genes (B2M, GAPDH and RPL13), that previous experiments showed to be RISC-associated but not miR-210 targets [15], were used for normalization.

### miRNA Blockade and Over-expression

Locked Nucleic Acid (LNA) oligonucleotides against miR-210 or a control scramble sequence (Exiqon) were transfected by siRNA Transfection Reagent (Santa Cruz), following manufacturer instructions, in 40% confluent HEK-293 (4×10^3^ cells/cm^2^) at the final concentration of 100 nM. After 16 hrs, cells were re-fed with fresh medium and experiments were performed 24 hrs later. miR-210 was also inhibited using a lentiviral vector that express a “sponge” reporter gene (see supplementary information). Transient miR-210 over-expression was obtained by transfection of pSUPER-pre-miR-210 [18] using Fugene6 (Roche). To obtain stable miR-210 over-expressing cells, HUVEC were infected by retroviral vectors bearing a pre-miR-210 sequence or a control scramble sequence, as previously described [18].

### Western Blotting

Cells were lysed in 2x Laemmli buffer and boiled for 5 minutes. Proteins were separated in SDS-polyacrylamide gels and transferred to nitrocellulose by standard procedures [20]. The following antibodies were used: ROD1 (F-30, Santa Cruz), α-tubulin (Ab-1, Oncogene research products). Expression levels were evaluated by densitometric analysis using a GS-710 scanner (Biorad) and Quantity One software. Protein expression was normalized for α-tubulin levels.

### Free Energy Calculation

The RNAhybrid tool (Bielefeld University Bioinformatics Server) was used to calculate the minimum free energy hybridization for short RNA duplexes.

### Statistical Analysis

Variables were analyzed by both Student’s t test and one way ANOVA. Unless differently specified, a p≤0.05 was deemed statistically significant. n = number of independent experiments. Values are expressed as ±standard error.

### Additional Methods

See Methods S1 for information on apoptosis analysis, lentiviral infection, luciferase activity assays, miRNA array, miRNA and mRNA quantification, miRNA target prediction and plasmids (additional methods).

## Results

### ROD1 is a miR-210-seedless Transcript Enriched in miR-210-containing-RISCs

**Figure 1:**
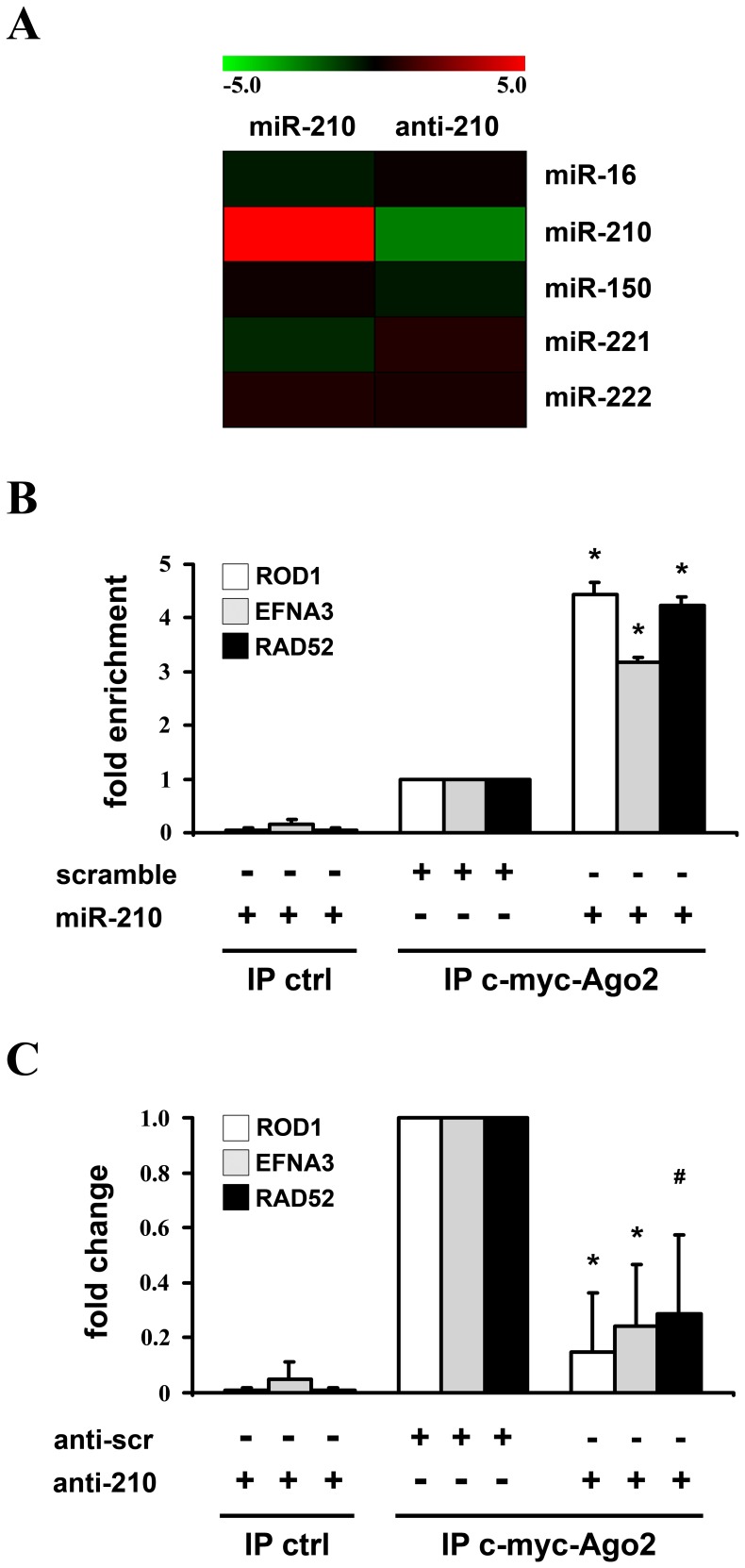
Increased or decreased miR-210 levels are associated to higher or lower ROD1 transcript levels in the RISC, respectively. HEK-293 were co-transfected with expression vectors for either miR-210 or a scramble sequence, and c-myc-Ago2 (A, miR-210 column and B). Alternatively, HEK-293 (4×10^3^/cm^2^) were co-transfected with anti-miR-210 or anti-scramble LNA-oligonucleotides and c-myc-Ago2 (A, anti-210 column and C). Then, c-myc antibody was used to immuno-precipitate c-myc-Ago2-containing complexes. A) miRNA levels in RISCs derived from transfected cells were assayed by qPCR. As expected, miR-210 was enriched or deprived in RISCs derived from cells in which miR-210 levels were up- or down-modulated, respectively, whereas other miRNAs did not show any significant modulation. Average values are expressed using a log2 scale. Green and red colors indicate down- or up-regulation, respectively (n = 3; p<0.01). B) ROD1 mRNA levels in RISCs derived from miR-210-enriched cells were assayed by qPCR. Background controls were represented by c-myc-immuno-precipitates derived from cells transfected with miR-210, but not mAgo2 (n = 7; *p<0.001). C) ROD1 mRNA levels in RISCs derived from miR-210-deprived cells were assayed by qPCR. Background controls were represented by c-myc-immuno-precipitates derived from cells transfected with anti-miR-210 LNA-oligonucleotides, but not mAgo2 (n = 3; *p<0.001; #p<0.01).

In a previous report, we identified a set of 62 miR-210 potential targets, selected for their reciprocal modulation with miR-210 [15]. Twenty of them, albeit inversely correlated to miR-210 (as summarized in Fig.S1), were not further analyzed since they did not display a “canonical” complementarity to miR-210 seed region. So, in order to experimentally test whether any of these genes was a seedless direct target, their over-representation in miR-210-enriched RISCs was assayed. To this aim, HEK-293 cells were co-transfected with expression vectors for miR-210 and c-myc-Ago2 (mAgo2), a core component of the RISC, yielding cells enriched of miR-210/mAgo2-containing RISC (Fig.1A). Then, we immuno-precipitated mAgo2 and evaluated the levels of co-immuno-precipitated mRNAs by qPCR. Background controls were represented by c-myc-immuno-precipitates derived from cells transfected with miR-210, but not mAgo2. Data were normalized against the average values of B2M, GAPDH and RPL13, that are RISC-associated but not miR-210 targets [15]. Six miR-210 known targets were used as positive controls. As expected, all of them were significantly enriched in immuno-precipitates of miR-210 over-expressing cells compared to cells transfected with a scramble sequence (Table S1). When miR-210 seedless transcripts were analyzed, to minimize the number of false positives we further considered only genes enriched significantly (p<0.01) more than 4 fold and confirmed by an independent primer couple (Table S1). According to this analysis, the only miR-210-seedless transcript enriched in miR-210-loaded RISC was ROD1. Fig.1B shows that ROD1 was as enriched as EFNA3 and RAD52, two well established miR-210 targets [8].

Next, we asked whether ROD1 bound to the RISC also in the presence of physiological miR-210 levels. To this aim, we set-up the reciprocal assay, hypothesizing that miR-210 blockade will lead to a decrease of miR-210 targets bound to RISC. Figure 1C shows that ROD1 was under-represented, as much as EFNA3 and RAD52, in immuno-precipitates derived from cells where mAgo2 was expressed and miR-210 was inhibited by a specific LNA-anti-miR-210 sequence (Fig.1A and Fig.S2A).

### Further Validation of ROD1 as a miR-210 Target

**Figure 2:**
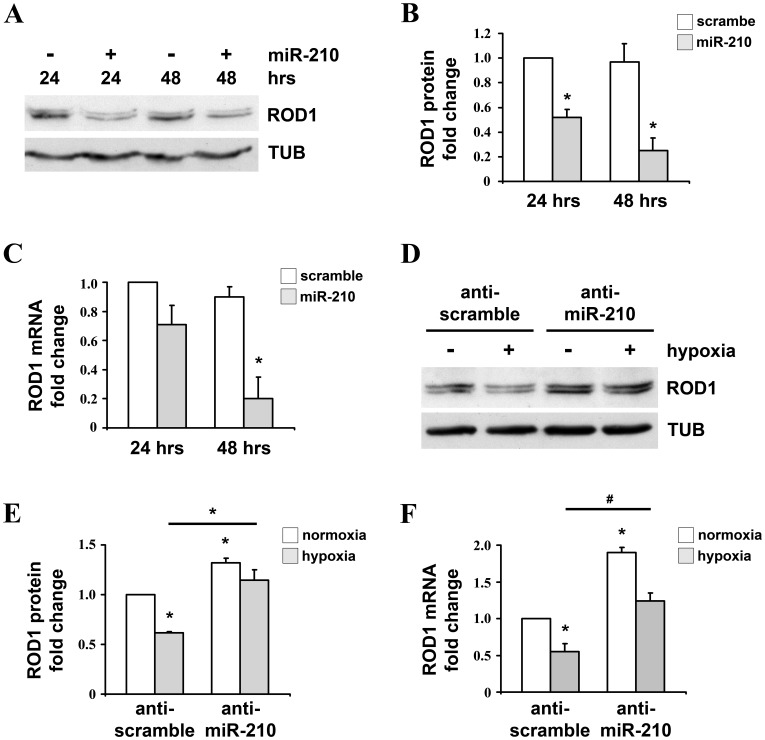
miR-210 and hypoxia decrease ROD1 expression. HEK-293 were transfected with plasmids encoding either miR-210 (miR-210) or a scramble sequence (scramble). Then, 24 and 48 hrs later, cells were collected and ROD1 protein and mRNA were assayed (A–C). Alternatively, HEK-293 were infected with lentiviruses expressing anti-scramble or anti-miR-210 sponges and, 24 hrs later, cells were exposed to hypoxia for further 72 hrs. Then cells were collected and ROD1 protein and mRNA were assayed (D-F). A) A representative western blot is shown; TUB: α-tubulin. B) The bar graph represents average ROD1 protein expression levels, measured by densitometric analysis and normalized for α-tubulin levels (n = 4; *p<0.001). C) ROD1 mRNA levels were measured by qPCR (n = 3; *p<0.001). D) A representative western blot is shown; TUB: α-tubulin. E) Expression levels of ROD1 protein were evaluated by densitometric analysis and normalized for α-tubulin protein levels (n-normoxia = 9; n-hypoxia = 5; *p<0.001). F) ROD1 mRNA levels were measured by qPCR (n = 3; *p<0.05; #p<0.005).

To further validate our results, protein and mRNA levels of ROD1 were evaluated after miR-210 modulation in HEK-293 cells. As shown in Fig.2A-C, ROD1 protein (A–B) and mRNA (C) were down-modulated following miR-210 over-expression. Then, we asked whether ROD1 was modulated in hypoxic conditions and if this modulation was miR-210 dependent. To this aim, miR-210 was down-modulated using retroviral vectors bearing a pre-miR-210 sequence or a control scramble sequence (Fig.S2B). We found that hypoxia down-modulated ROD1, both at protein and mRNA level, and that miR-210-inhibition rescued this regulation (Fig.2D-F).

### Confirmation of ROD1 as miR-210 Seedless Transcript

Six ROD1 transcript variants have been annotated (NCBI) and none of these ROD1 isoforms displayed a miR-210-seed complementarity sequence, even though all the parts (i.e. 5′UTR, *cds* and 3′UTR) of each transcript were analyzed. We asked if any minor (less represented) isoform contained a putative miR-210-seed binding site. Out of 490 published Expressed Sequence Tags (NCBI), only 4 displayed a miR-210-seed complementary sequence (Clone IDs: BG026147; BE617510, BG256179, BG387676). However, for all of them, the EST portion containing the miR-210-seed complementarity did not align to any region of the genome, likely representing cloning or sequencing artifacts. In conclusion, while we cannot formally exclude that a minor ROD1 isoform may be a canonical miR-210 target, this is an unlikely and rare event.

### ROD1 is not a Predicted Target of miRNAs Modulated by miR-210

A possible explanation of the results obtained in the RISC-IP and miR-210 modulation experiments (Fig.1–2) was that miR-210 induced the expression of a second miRNA that, in turn, targeted ROD1 with a canonical mechanism. In order to address this issue, HUVEC over-expressing pre-miR-210 or a scramble sequence were generated by retroviral infection, yielding a selected population that expressed mature miR-210 levels comparable with those observed in hypoxic cells [18]. miRNA expression profiles were then measured and miRNAs increased upon miR-210 up-regulation were identified (Table 1, see Table S2 for the complete miRNA dataset). Target prediction analysis was then conducted for each of these miRNAs using six among the most widely used algorithms. Results showed that none of the miR-210-induced miRNAs was predicted to target ROD1.

**Table 1:** miRNAs modulated by miR-210.

miRNA		Fold change	P.Value	adj.P.Val
hsa-let-7d*		1.4	0.0001	0.0053
hsa-miR-187*		1.9	0.0000	0.0010
hsa-miR-18b		0.7	0.0001	0.0053
hsa-miR-195*		1.6	0.0001	0.0053
hsa-miR-215		1.5	0.0003	0.0094
hsa-miR-28-5p		0.7	0.0001	0.0053
hsa-miR-298		1.5	0.0002	0.0070
hsa-miR-301a		0.6	0.0000	0.0022
hsa-miR-32*		1.7	0.0001	0.0061
hsa-miR-526b		1.6	0.0002	0.0063
hsa-miR-576-3p		1.7	0.0001	0.0053
hsa-miR-583		1.6	0.0001	0.0053
hsa-miR-602		1.4	0.0003	0.0079
hsa-miR-620		1.8	0.0001	0.0053
hsa-miR-635		1.5	0.0001	0.0053
hsa-miR-640	1.8		0.0000	0.0048
hsa-miR-877		1.4	0.0003	0.0079
hsa-miR-920		1.8	0.0000	0.0048

P.Value (moderated t-statistic) adj.P.Val (p-value adjusted for False Discovery Rate).

### ROD1 is Directly Inhibited by miR-210 Through an Atypical Centered Pairing

**Figure 3:**
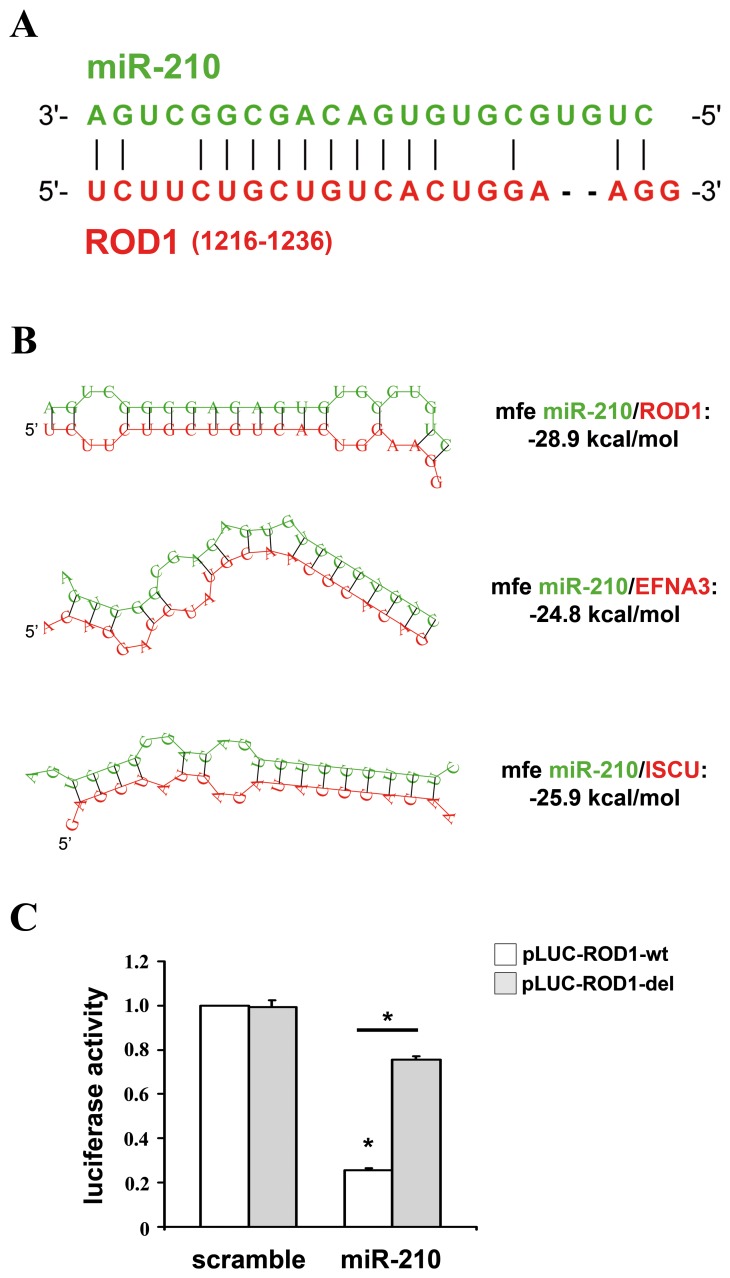
miR-210 targets ROD1 directly. A) miR-210 (green) and ROD1 (red) complementarity. ROD1 transcript variant 6 (NM_001244898) is the longest ROD1 isoform and it was used for base numeration. B) RNA-duplexes prediction between miR-210 (green) and ROD1, EFNA3 and ISCU (red). Their calculated thermodynamic stabilities are indicated (mfe = minimum free energy). The mRNA sequences displayed are the following (gene name, RefSeq, bases, location): ROD1-isoform 6, NM_001244898, 1216–1236, cds; EFNA3, NM_004952,798–804, 3′UTR; ISCU, NM_014301, 111–118, 3′UTR. C) HEK-293 were transfected with firefly luciferase constructs that contain either an intact (pLUC-ROD1-wt) or a mutated miR-210 binding site (pLUC-ROD1-del). pLUC plasmids were co-transfected with a plasmid encoding renilla luciferase along with a plasmid encoding either miR-210 (miR-210) or a scramble sequence (scramble). Firefly luciferase values were normalized according to renilla luciferase activity (n = 4; *p<0.001).

We tried to identify atypical miR-210 binding sites in ROD1. A miRNA target prediction analysis was computed using Rna22 algorithm. Rna22 requires the presence of a seed-complementarity, but allows atypical pairings (i.e. G:U) and searches for miRNA binding sites in both the 3′UTR and the *cds* of the sequence of interest. Rna22 prediction identified 3 miR-210 potential binding sites in ROD1. These regions were cloned in luciferase reporter constructs, assayed for the direct binding of miR-210, but did not show any miR-210-dependent modulation (Fig.S3). Next, we tried a low-stringency pairing search in the whole sequence of ROD1 and we identified a putative miR-210 binding site in the *cds* of ROD1 (Fig.3A). This complementary pairing involved eight consecutive bases in the central portion of miR-210, did not involve the seed region and was present in all the six transcript variants of ROD1. RNA complementarity was further extended to 10 consecutive bases, allowing one G:U pairing, and 2 nucleotide-long complementarities were also present at both the 5′- and the 3′-end of miR-210 (Fig.3A). The thermodynamic stability of the putative RNA-duplex was computed using RNAhybrid tool, that indicated an hybridization minimum free energy of -28.9 kcal/mol (Fig.3B). The minimum free energy of miR-210/ROD1 interaction was comparable to those predicted for EFNA3 and ISCU, two well established miR-210 targets [8]. In order to investigate if the identified ROD1 region was involved in the inhibition by miR-210, we cloned the miR-210-pairing site and the surrounding sequences, downstream of a luciferase open reading frame. A mutant of miR-210/ROD1 pairing sequence was used as control and the luciferase activity of these constructs was evaluated following the over-expression of either miR-210 or a scramble sequence. Results in Fig.3C show that miR-210 inhibited the reporter construct containing an intact miR-210 binding site (pLUC-ROD-wt), but not the reporter with the deletion of the complementary nucleotides (pLUC-ROD-del). These data demonstrated that ROD1 is directly targeted by miR-210.

### Other PTB Family Members are not Modulated by miR-210

ROD1 is a paralog gene of the polypyrimidine tract binding proteins PTBP1 and PTBP2 [21]. For this reason, it was recently included in the PTBP family and named PTBP3. Although miR-210 complementary sequence was not present in ROD1 paralogs, we tested if also PTBP1 and PTBP2 could be modulated by miR-210. RISC-IP experiments demonstrated that neither PTBP1 nor PTBP2 was enriched in miR-210-containing-RISCs (Fig.S4A). Moreover, PTBP1 and PTBP2 mRNA levels were not modulated upon miR-210 over-expression (Fig.S4B-C). Thus, at least in the adopted experimental conditions, ROD1 paralogs are not regulated by miR-210.

### ROD1 Levels Regulate Cell Survival

**Figure 4:**
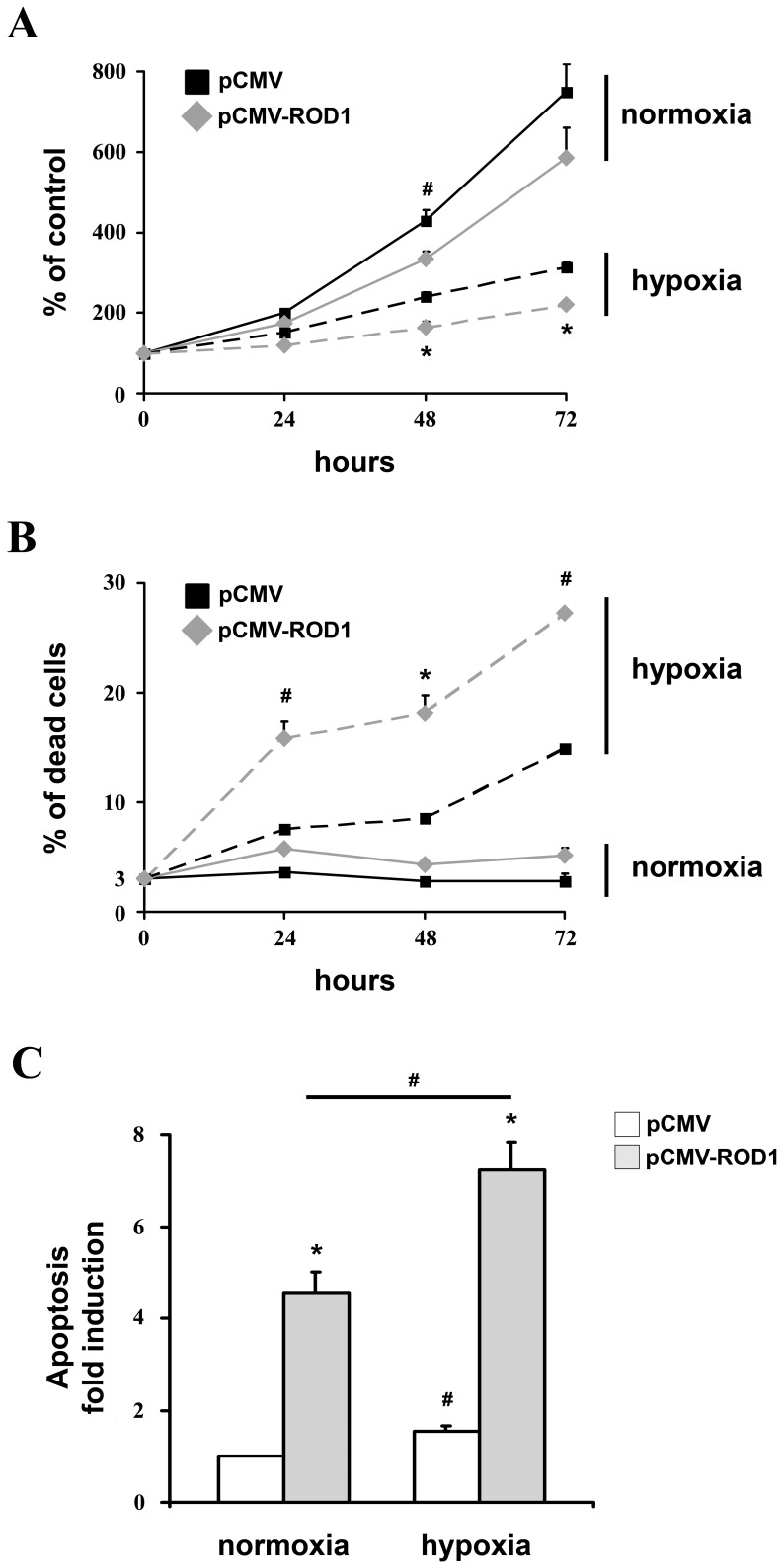
The override of ROD1 down-modulation by hypoxia increased apoptosis. HEK-293 were transfected with plasmids encoding ROD1 (pCMV-ROD1) or with vector alone (pCMV) and the next day were exposed to 1% hypoxia for the indicated time. A) HEK-293 growth curve. Data are expressed as % of T0 normoxic control. Significant differences between pCMV-ROD1 and pCMV transfected cells in the same experimental condition are indicated (n = 5–11; *p<0.001; #p<0.008). B) Cell death assessed by Trypan blue exclusion assay. Data are expressed as % of dead cells for each experimental point. Differences between hypoxic cells transfected with either pCMV-ROD1 or pCMV are statistically significant (n = 5–11; *p<0.001; #p<0.008). C) Transfected cells were exposed to hypoxia for 48 hrs and apoptosis was measured assessing the apoptotic fragmentation of cytoplasmic DNA (n = 3; *p<0.007; #p<0.03).

We and others demonstrated that the anti-apoptotic activity of miR-210 is a fundamental adaptive response to hypoxia [8]. Thus, we investigated whether ROD1 targeting is necessary for miR-210 action. We tested whether the override of ROD1 down-modulation by hypoxia decreased cell survival. To this aim, ROD1 was over-expressed in HEK-293 that were subsequently shifted to hypoxic conditions, rescuing the miR-210-dependent ROD1 down-modulation induced by hypoxia (Fig.S5). Growth curves of transduced cells demonstrated that ROD1 expression inhibited cell proliferation, both in the presence and in the absence of hypoxia (Fig.4A). However, when cell survival was measured, we found that ROD1 expression dramatically increased hypoxia-induced cell death (Fig.4B) and apoptosis (Fig.4C). These data indicate that ROD1 expression regulates cell survival and that its repression is involved in miR-210 anti-apoptotic function. Next, miR-210 was down-modulated in cells over-expressing ROD1. As expected, miR-210 blockade decreased cell proliferation and increased cell death (Fig.S6). Moreover, miR-210 inhibition in ROD1 over-expressing cells further decreased cell proliferation (Fig.S6A), increasing cell death (Fig.S6B) and apoptosis (Fig.S6C). These data suggest that the anti-apoptotic activity of miR-210 is also mediated by other targets besides ROD1.

## Discussion

In this study, we identified ROD1 as a seedless target of the hypoxia-induced miR-210. ROD1 emerged as the only direct target among 20 seedless genes displaying an inverse correlation with miR-210 expression [15]. The low rate of validation among seedless genes confirmed the prevalence of the seed rule as major mechanism for miRNA-mediated inhibition of mRNAs. In fact, our previous study showed that 40% of the inversely correlated genes that contained a putative miR-210-seed binding sequence were enriched in the miR-210-containing RISC [15]. However, it is worth noting that in our search of seedless targets we applied a more stringent cut-off in order to minimize the number of false positives. Indeed, FXR1 (Fragile X mental retardation, autosomal homolog 1) and LANCL1 (LanC lantibiotic synthetase component C-like 1) displayed a small but reproducible enrichment in miR-210-containing RISCs (Table S2), suggesting that FXR1 and LANCL1 may be miR-210 seedless targets as well. Additional experiments are required to confirm this hypothesis. Our data are in agreement with other reports in which unbiased experimental approaches identified a sub-set of potential miRNA seedless targets [22], [23]. The amount of seedless targets was measured by Chi and colleagues that, through the purification of the mRNAs bound to the native RISC followed by high-throughput sequencing, identified 27% of non-canonical miRNA targets in the mouse brain [24].

We considered the possibility that ROD1 was an indirect miR-210 target. Indeed, one can envision a model where a miR-210-induced miRNA targets ROD1 and recruits it to the RISC. For instance, it was recently demonstrated that ROD1 is a target of miR-499 [25]. However, miR-499 is expressed mainly in the cardiac and skeletal muscles; moreover, miR-499 was never found to be induced by hypoxia [10], [26], nor by miR-210 expression.

When we tried to identify a miR-210 binding site in ROD1, a region with partial pairing to miR-210 was discovered in the *cds*. Luciferase reporter assays formally demonstrate that miR-210 inhibits ROD1 by the direct binding to this sequence. The identified miR-210/ROD1 pairing was similar to the “centered pairing” recently described by Bartel and collaborators [6], since it involved 10 consecutive bases in the central portion of miR-210 (nt 9–18). Because of the location and extent of their base pairing, centered sites occupy a intermediate position between partial pairings, involving the seed sites and associated to translation inhibition, and extensive pairings, associated with RNA interference-type degradation. Specifically, complementarity with the 9–12 nucleotides of the miRNA is known to activate the phosphodiesterase activity of Ago2, inducing the cleavage and degradation of the target mRNA [1]. Accordingly, ROD1 mRNA was strongly down-modulated upon miR-210 over-expression (Fig.2C). On the other hand, the ratio between the RISC activities of RNA cleavage and repression of translation is regulated by Mg^2+^ concentrations [6]. Shin et al. demonstrated that the centered pairings favor the inhibition of translation in the presence of high intracellular Mg^2+^ concentrations [6]. Therefore, in physiological conditions, miR-210 will likely inhibit ROD1 by both mechanisms.

Other atypical miRNA binding patterns were recently described [3], [7]. Specifically, in a study focused on miR-24-dependent inhibition of cell proliferation, 7 seedless targets of miR-24 were identified experimentally [5]. It is remarkable that miR-24 binds to the mRNA of these genes with a variety of patterns, confirming that the rules governing miRNA activity are far from being fully elucidated.

Finally, the biological significance of ROD1 targeting was evaluated. The results highlighted the importance of a tight control of ROD1 levels for cell survival in hypoxia. In fact, we found that the rescue of miR-210-dependent down-modulation of ROD1 observed in hypoxic cells increased apoptosis. These data indicate that miR-210 is part of the molecular mechanisms fine tuning ROD1 expression. In keeping with this interpretation, ROD1 levels, that are induced in a mouse model of hindlimb ischemia, are further increased upon miR-210 blockade (G. Zaccagnini and F. Martelli, in preparation).

Correlative evidences also indicate that miR-210 plays a pivotal role in ROD1 regulation. A negative activity of ROD1 in the differentiation control of mammalian cells is emerging. Specifically, ROD1 over-expression blocks the erythroid differentiation of a human leukemia cell line [27], [28]. Moreover, ROD1 inhibition by miR-499 enhances cardiomyogenesis *in vitro* and after infarction *in vivo* [25]. miR-210, on the contrary, is induced during differentiation in many cellular contexts [10], including erythroid differentiation [29], [30] and human embryonic stem cells differentiation to endothelium [31]. In this scenario, ROD1 targeting could be also important for miR-210 pro-angiogenic activity [18].

## Supporting Information

Figure S1 **miR-210-seedless genes modulated by miR-210.** Heat map representing mRNAs down-modulated by miR-210 over-expression (miR-210 column) that displayed an inverse modulation following miR-210 inhibition (anti-210 column) in HUVEC. The only exception is constituted by CTSB, NDUFV2, TCEB2 and VIM that were measured at protein level in miR-210 over-expressing HUVEC. Average values are expressed as fold change. Green and red colors indicate down- or up-regulation, respectively, while gray indicates missing data (n = 3; p<0.005 for transcriptomics and p<0.05 for proteomics).
(TIF)

Figure S2 **Quantification of miR-210 inhibition.** A) HEK-293 were transfected with anti-miR-210 or anti-scramble LNA-oligonucleotides. miR-210 levels were assayed 40 hrs later, confirming an efficient miR-210 knock-down (n = 3; *p<0.002). B) HEK-293 were infected with lentiviruses expressing anti-scramble or anti-miR-210 sponges. miR-210 levels were assayed 72 hrs later, confirming an efficient miR-210 knock-down (n = 3; *p<0.03).
(TIF)

Figure S3 **Prediction of the putative binding sites between miR-210 and ROD1 according to the Rna22 algorithm.** ROD1 transcript variant 6 (NM_001244898) is the longest ROD1 isoform and it was used for base numeration.
(TIF)

Figure S4 **PTBP1 and PTBP2 paralogs are not miR-210 targets.** A) HEK-293 were co-transfected with expression vectors for either miR-210 or a scramble sequence and c-myc-Ago2 (mAgo2). Then, c-myc antibody was used to immuno-precipitate the miR-210/mAgo2-containing complexes. Background controls were represented by c-myc-immuno-precipitates derived from cells transfected with miR-210 but not mAgo2. Whereas ROD1 was enriched in the immune-precipitates of the mir-210-loaded RISC, PTBP1 and PTBP2 did not show any significant modulation (n = 3; *p<0.001). B-C) HEK-293 were transfected with plasmids encoding either miR-210 (miR-210) or a scramble sequence (scramble). Then, 24 and 48 hrs later, cell extracts were derived and the and mRNA levels of PTBP1 (B) and PTBP2 (C) were assayed by qPCR (n = 3).
(TIF)

Figure S5 **Override of ROD1 down-modulation by hypoxia.** HEK-293 were transfected with plasmids encoding ROD1 (pCMV-ROD1) or with vector alone (pCMV) and the next day were exposed to 1% hypoxia for the indicated time. miR-210 and ROD1 levels were assayed by qPCR. Average values are expressed using a log2 scale. Green and red colors indicate down- or up-regulation, respectively (n = 3; p<0.01).
(TIF)

Figure S6 **miR-210 inhibition in ROD1 over-expressing cells further decreases cell survival.** HEK-293 (4×10^3^/cm^2^) were co-transfected with anti-miR-210 or anti-scramble LNA-oligonucleotides and pCMV-ROD1 (ROD1) or vector alone (pCMV). A) HEK-293 growth curve. Data are expressed as % of T0 normoxic control. Significant differences between anti-miR-210 and anti-scramble transfected cells in the same experimental condition are indicated (n = 3; *p<0.001; #p<0.005). B) Cell death assessed by Trypan blue exclusion assay. Data are expressed as % of dead cells for each experimental point. Significant differences between anti-miR-210 and anti-scramble transfected cells in the same experimental condition are indicated (n = 3; *p<0.01). C) After 48 hrs of transfection, apoptosis was measured assessing the apoptotic fragmentation of cytoplasmic DNA (n = 3; *p<0.007; #p<0.03).
(TIF)

Table S1 RISC-IP of miR-210-seedless genes.
(DOC)

Table S2 miRNAs array upon miR-210 over-expression.
(XLSX)

Table S3 Primers for SYBR-GREEN Real Time PCR.
(DOC)

Methods S1 **Supplementary methods.**
(DOC)
