# Supplementary figures and images for: ROD1 Is a Seedless Target Gene of Hypoxia-Induced miR-210
Source: PLoS One. 2012 Sep 14;7(9):e44651. doi: 10.1371/journal.pone.0044651 (PMC3443109; doi:10.1371/journal.pone.0044651)

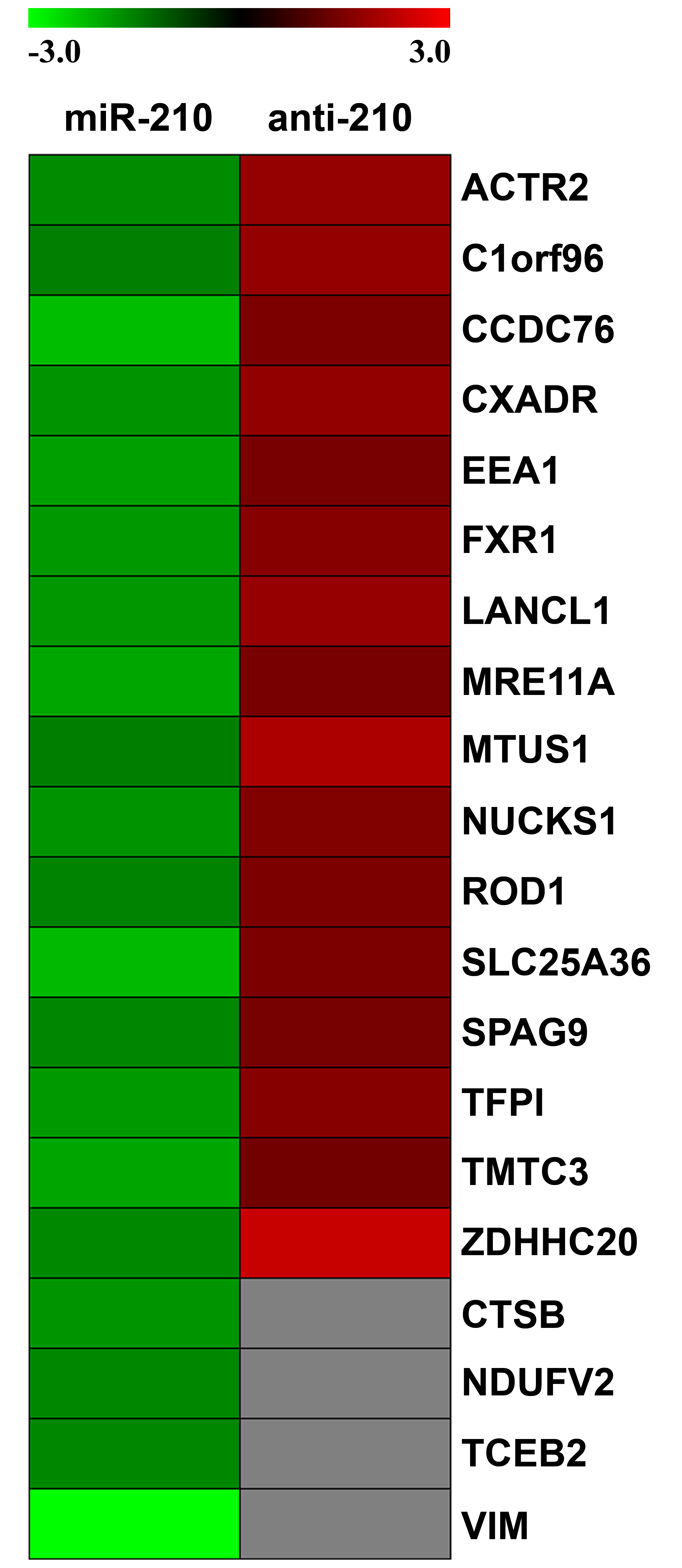

Supplement: Figure S1 — miR-210-seedless genes modulated by miR-210. Heat map representing mRNAs down-modulated by miR-210 over-expression (miR-210 column) that displayed an inverse modulation following miR-210 inhibition (anti-210 column) in HUVEC. The only exception is constituted by CTSB, NDUFV2, TCEB2 and VIM that were measured at protein level in miR-210 over-expressing HUVEC. Average values are expressed as fold change. Green and red colors indicate down- or up-regulation, respectively, while gray indicates missing data (n = 3; p<0.005 for transcriptomics and p<0.05 for proteomics). (TIF) [file pone.0044651.s001.tif]

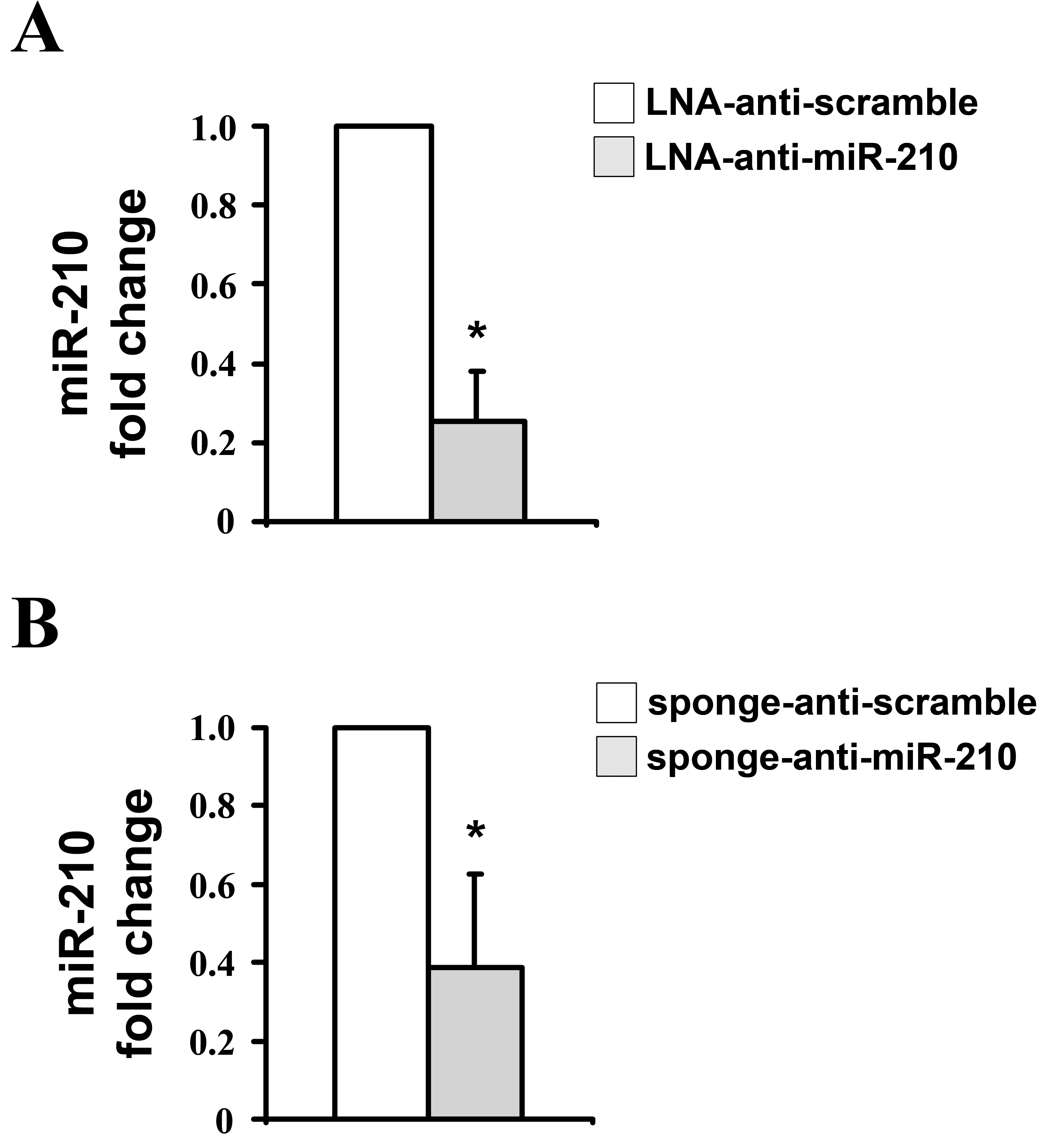

Supplement: Figure S2 — Quantification of miR-210 inhibition. A) HEK-293 were transfected with anti-miR-210 or anti-scramble LNA-oligonucleotides. miR-210 levels were assayed 40 hrs later, confirming an efficient miR-210 knock-down (n = 3; *p<0.002). B) HEK-293 were infected with lentiviruses expressing anti-scramble or anti-miR-210 sponges. miR-210 levels were assayed 72 hrs later, confirming an efficient miR-210 knock-down (n = 3; *p<0.03). (TIF) [file pone.0044651.s002.tif]

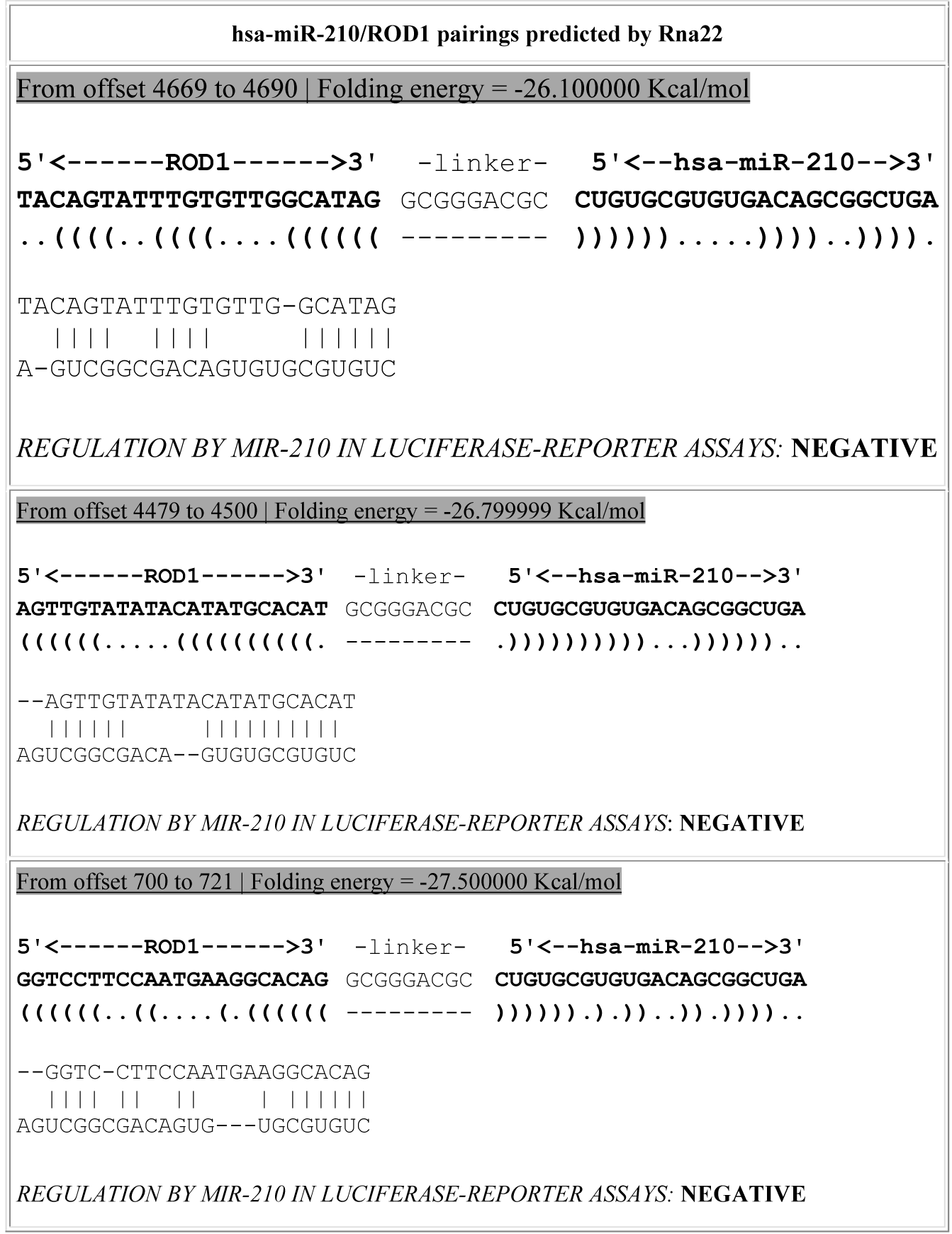

Supplement: Figure S3 — Prediction of the putative binding sites between miR-210 and ROD1 according to the Rna22 algorithm. ROD1 transcript variant 6 (NM_001244898) is the longest ROD1 isoform and it was used for base numeration. (TIF) [file pone.0044651.s003.tif]

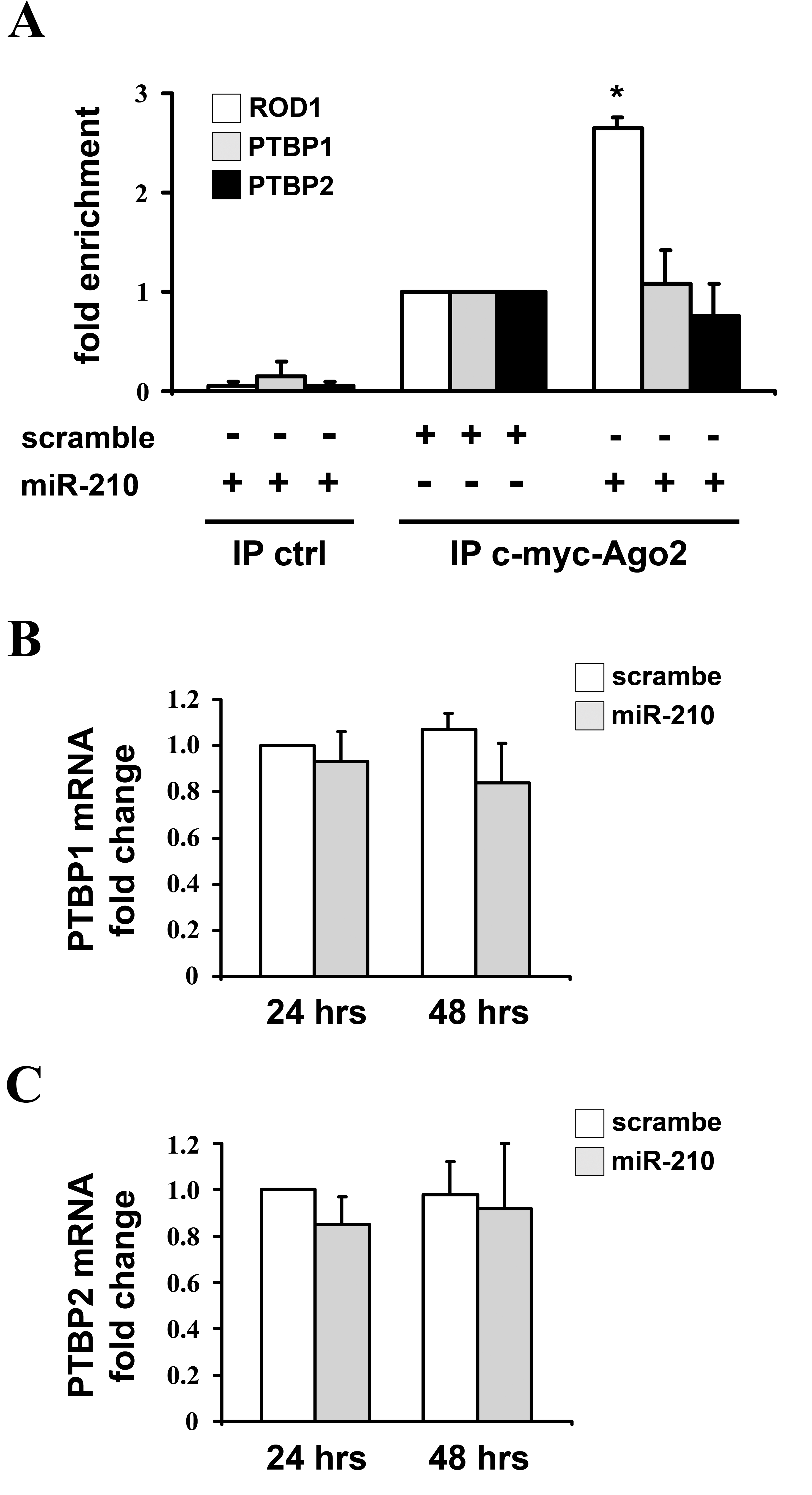

Supplement: Figure S4 — PTBP1 and PTBP2 paralogs are not miR-210 targets. A) HEK-293 were co-transfected with expression vectors for either miR-210 or a scramble sequence and c-myc-Ago2 (mAgo2). Then, c-myc antibody was used to immuno-precipitate the miR-210/mAgo2-containing complexes. Background controls were represented by c-myc-immuno-precipitates derived from cells transfected with miR-210 but not mAgo2. Whereas ROD1 was enriched in the immune-precipitates of the mir-210-loaded RISC, PTBP1 and PTBP2 did not show any significant modulation (n = 3; *p<0.001). B-C) HEK-293 were transfected with plasmids encoding either miR-210 (miR-210) or a scramble sequence (scramble). Then, 24 and 48 hrs later, cell extracts were derived and the and mRNA levels of PTBP1 (B) and PTBP2 (C) were assayed by qPCR (n = 3). (TIF) [file pone.0044651.s004.tif]

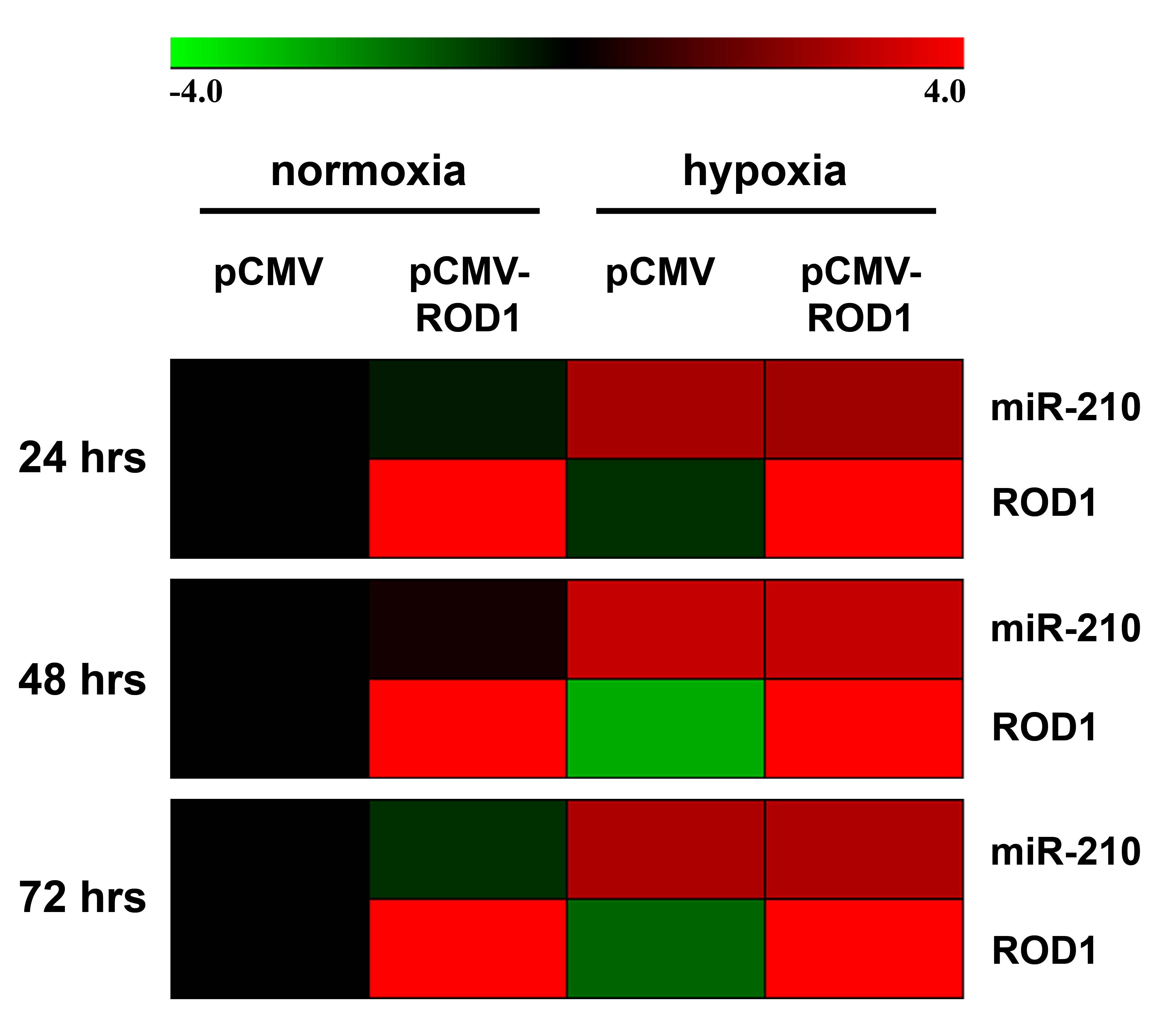

Supplement: Figure S5 — Override of ROD1 down-modulation by hypoxia. HEK-293 were transfected with plasmids encoding ROD1 (pCMV-ROD1) or with vector alone (pCMV) and the next day were exposed to 1% hypoxia for the indicated time. miR-210 and ROD1 levels were assayed by qPCR. Average values are expressed using a log2 scale. Green and red colors indicate down- or up-regulation, respectively (n = 3; p<0.01). (TIF) [file pone.0044651.s005.tif]

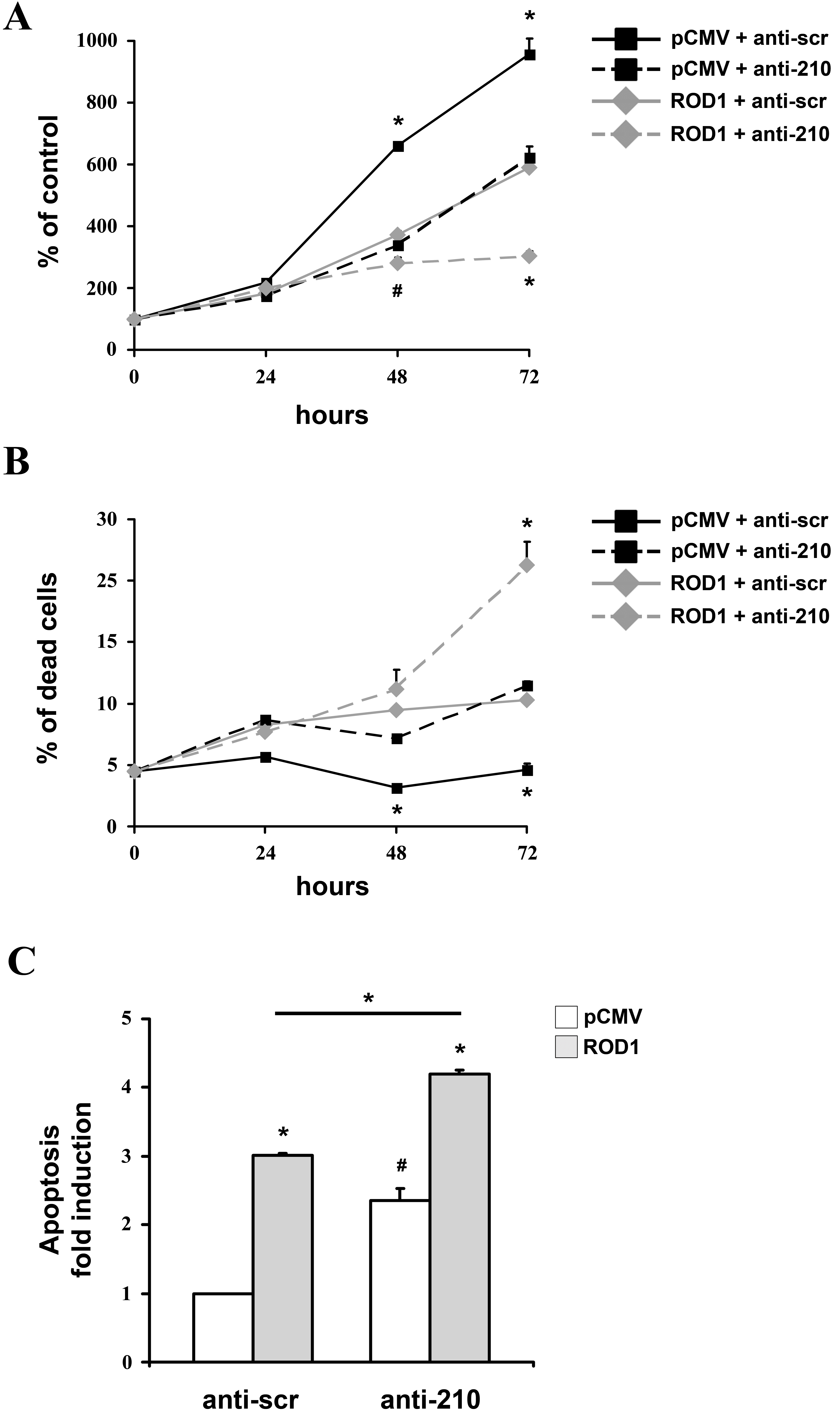

Supplement: Figure S6 — miR-210 inhibition in ROD1 over-expressing cells further decreases cell survival. HEK-293 (4×10^3^/cm2) were co-transfected with anti-miR-210 or anti-scramble LNA-oligonucleotides and pCMV-ROD1 (ROD1) or vector alone (pCMV). A) HEK-293 growth curve. Data are expressed as % of T0 normoxic control. Significant differences between anti-miR-210 and anti-scramble transfected cells in the same experimental condition are indicated (n = 3; *p<0.001; #p<0.005). B) Cell death assessed by Trypan blue exclusion assay. Data are expressed as % of dead cells for each experimental point. Significant differences between anti-miR-210 and anti-scramble transfected cells in the same experimental condition are indicated (n = 3; *p<0.01). C) After 48 hrs of transfection, apoptosis was measured assessing the apoptotic fragmentation of cytoplasmic DNA (n = 3; *p<0.007; #p<0.03). (TIF) [file pone.0044651.s006.tif]
