# Supplementary material for: ROD1 Is a Seedless Target Gene of Hypoxia-Induced miR-210
Source: PLoS One. 2012 Sep 14;7(9):e44651. doi: 10.1371/journal.pone.0044651 (PMC3443109; doi:10.1371/journal.pone.0044651)
Supplement: Table S1 — RISC-IP of miR-210-seedless genes. (DOC) [file pone.0044651.s007.doc]

**SUPPLEMENTARY TABLE 1: RISC-IP of miR-210-seedless genes**
