# Supplementary material for: ROD1 Is a Seedless Target Gene of Hypoxia-Induced miR-210
Source: PLoS One. 2012 Sep 14;7(9):e44651. doi: 10.1371/journal.pone.0044651 (PMC3443109; doi:10.1371/journal.pone.0044651)
Supplement: Table S3 — Primers for SYBR-GREEN Real Time PCR. (DOC) [file pone.0044651.s009.doc]

**SUPPLEMENTARY TABLE 3: primers for SYBR-GREEN Real Time PCR**

| **GENE** | **DESCRIPTION** | **FORWARD_1** | **REVERSE_1** | **FORWARD_2** | **REVERSE_2** |
| --- | --- | --- | --- | --- | --- |
| ACTR2 | ARP2 actin-related protein 2 homolog (yeast) | gcggtggctgtaggttgt | gcctgcatatccacacttcac | atgtagccatccaggcagtt | gatgagggagagaaaagccttc |
| APC | adenomatosis polyposis coli down-regulated 1 | gcatggaccaggacaaaaat | gaacacacacagcaggacaga |  |  |
| ATP11C | ATPase, Class VI, type 11C | tgcatgtcagtatgcagatgg | gccaactcgtttctcttctcc |  |  |
| B2M | Beta-2-microglobulin | ttctggcctggaggctatc | tcaggaaatttgactttccattc |  |  |
| C1orf96 | chromosome 1 open reading frame 96 | ttttgagcactgcattcacttt | gcctgggctttgtgattatg |  |  |
| CCDC76 | coiled-coil domain containing 76 | gctgagggtagatgcggtta | cgagcatcttcttcctccat | gattctgcaaccaagcacct | tcaacaaagcatcttcttggac |
| CTSB | Cathepsin B precursor | tggagggagctttctctgtg | tgacgtgttggtacactcctg | cccaccatcaaagagatcaga | atgcagatccggtcagagat |
| CXADR | coxsackie virus and adenovirus receptor | atgaaaaggaagttcatcacgata | tgtagcttctggcagtggac | gccttgagatctgtggtggt | gaaaaatatcacctgggaactatga |
| E2F3 | E2F transcription factor 3 | atatccctaaacccgcttcc | tggtcctcagtctgctgtaaga |  |  |
| EEA1 | early endosome antigen 1 | tcaattaaatgaaagcaaggagaa | ttcggttttggctttaatctg | catgttaaggaggattttacagagg | tgctgatgaatctaaatcagaacc |
| EFNA3 | ephrin-A3 | ggatgaaggtgttcgtctgc | tctctccctcaaagtcttcca |  |  |
| FXR1 | fragile X mental retardation, autosomal homolog 1 | acagctgcgacagattggtt | tcagaggggttagacagctca | agaacgccaggttccattta | atggctcttggtcatttgct |
| GAPDH | Glyceraldehyde-3-phosphate dehydrogenase | agccacatcgctcagacac | gcccaatacgaccaaatcc |  |  |
| LANCL1 | LanC lantibiotic synthetase component C-like 1 (bacterial) | tggcctggctggaatttat | cttcccttggctcacttgaa | ccgaaggctactttgatgct | ttggtcaagcgttgtgagaa |
| MRE11A | MRE11 meiotic recombination 11 homolog A (S. cerevisiae) | aagatgatgaagtccgtgagg | gaagcagactcctctgactgagat | tttcaggcatagagaacaaaagg | tgaaggctttgtgataagtttcc |
| MTUS1 | mitochondrial tumor suppressor 1 | ccaagtttgaggcattgacag | ttgtgttgtttcagtgcttcct | acctccggggagagctagt | ggacgaatgcttcatacactgtt |
| NDUFV2 | NADH-ubiquinone oxidoreductase 24kDa subunit | agctcaaggctggcaaaat | ttgggtggttcagtcaaaga | ctggggaagacatgtaaggaa | ggttattctcaggagtatctctgtgc |
| NUCKS1 | nuclear casein kinase and cyclin-dependent kinase substrate 1 | gatgtgggcagtgaggaag | atcgctgccggaatcttt | gaggaggatgaggcacca | tcatcttccattaggaaatcttca |
| RAD52 | RAD52 homolog (S. cerevisiae) | tctttggagaaggcaaggaa | ccaagtgcattcccaaaact |  |  |
| ROD1 | ROD1 regulator of differentiation 1 (S. pombe) | ccattggatttcctcaagcta | aggtgattgtgagaggaccaa | gctgacccagtaaatgcaca | cagagtgcagcatgcattataga |
| RPL13 | Ribosomal protein L13 | ggagtaccgctccaaactca | ggtggccagtttcagttctt |  |  |
| SLC25A36 | Solute carrier family 25, member 36 | acacgctggtgcatctgtt | ccagtggacatgtcagaatagc | cagattttgtgggaatgatgc | tccctcttcacgtagtcttgttc |
| SPAG9 | sperm associated antigen 9 | ccatcccattgacagaaaca | tgtccctggagtcactttatca | tggctcaggcctaataggag | aacttcccgacccattcct |
| TCEB2 | Transcription elongation factor B polypeptide 2 | gcttcaccagtcaaacagca | gcctcaaaggtgtcatctgc | atggccaagagcagaaacac | gttccctcgttgaacatgc |
| TFPI | tissue factor pathway inhibitor (lipoprotein-associated coagulation inhibitor) | gcctgggcaatatgaacaat | ccacctggaaaccattcg | catgcactttgggcttctg | ggtggcaactccgtatctgt |
| TMTC3 | transmembrane and tetratricopeptide repeat containing 3 | ccgacttcaagcaggcttac | gcttcctttgctttaagaggttt | gaccatgactgtggtttcagtg | gcacttctggacaaaaactgg |
| VIM | Vimentin | aaagtgtggctgccaagaac | agcctcagagaggtcagcaa | gaccagctaaccaacgacaaa | gaagcatctcctcctgcaat |
| XIST | X (inactive)-specific transcript | ggctccaaattaatcatatctttcc | aacaacaagcctattcttctgagg |  |  |
| ZDHHC20 | zinc finger, DHHC-type containing 20 | cgcttgttggacagtgaatc | tgcaaatgaaaattgaaaatacca | aaatggtttctctcttggatgc | ggaaaactgcaaccatcacc |
