# Supplementary material for: ROD1 Is a Seedless Target Gene of Hypoxia-Induced miR-210
Source: PLoS One. 2012 Sep 14;7(9):e44651. doi: 10.1371/journal.pone.0044651 (PMC3443109; doi:10.1371/journal.pone.0044651)
Supplement: Methods S1 — Supplementary methods. (DOC) [file pone.0044651.s010.doc]

**SUPPLEMENTARY METHODS**

**Apoptosis analysis.** Apoptosis was assessed by measuring the amount of nucleosomes generated during the apoptotic fragmentation of cellular DNA by Cell Death Detection Elisa (Roche), according to manufacturer instructions.

**Lentiviral infection.** Purified lentiviruses were produced by the virus production facility of the Istituto Dermopatico dell'Immacolata IRCCS, Roma, Italy. HEK-293 were infected as previously described [1]. Briefly, cells were incubated for 2 hrs with 10μl/p6 dish of purified lentiviruses and then were allowed to recover in complete fresh medium for an additional 24 hrs.

**Luciferase activity assay.** The rationale of this assay is based on the translational inhibition of the firefly luciferase gene by the cloning of miRNA-targeted sequences in its 3’UTR. HEK-293 cells, plated in p3 dishes, were transfected with 30 ng of pLUC, pLUC-ROD1-wt or pLUC-ROD1-del (see Plasmid section), 1μg of pSUPER-pre-miR-210 or pSUPER-scramble, and 25 ng of pRL-null renilla luciferase. Cellular extracts were tested with Dual Luciferase Assay (Promega) according to the manufacturer instructions, using a EG&G Berthold MicroLumat Plus LB96V, 48 hrs after transfection. Values were normalized according to renilla luciferase activity.

**miRNA array.** Total RNA was extracted using TRIzol (Invitrogen). Two-color hybridization was performed using miRCURY LNA microRNA Arrays (v.10.0, EXIQON). The obtained data were analyzed using the Limma package from the Bioconductor Project, subjecting the arrays to locally weighted scatterplot smoothing (Loess). miRNAs with spots showing less than 1.5 times median signal intensity were not considered for subsequent analysis. The complete dataset of our study is available on the NCBI Gene Expression Omnibus data base, GEO series GSE35365.

**miRNA and mRNA quantification.** Total RNA was extracted using TRIzol (Invitrogen). miRNA levels were analyzed using the TaqMan quantitative real-time PCR (qPCR) method (1 ng/assay) and quantified with the ABI Prism 7000 SDS (Applied Biosystems). Primers for miRNA assay, reagents for reverse transcriptase and qPCR reactions were all obtained from Applied Biosystems. Different samples were normalized to miR-16 expression. mRNAs levels were analyzed using the SYBR-GREEN qPCR method (5 ng/assay, Qiagen) and quantified with ABI Prism 7000 SDS (Applied Biosystems). Relative expression was calculated using the comparative Ct method (2^–[delta][delta]Ct^) [2]. Unless differently specified, mRNA expression was normalized against the average values of B2M and RPL13. See Supplementary Table III for details about primers. Heat maps were generated using Genesis software (version 1.7.5, Graz University of Technology, Institute for Genomics and Bioinformatics).

**miRNA target prediction.** Bioinformatic prediction of target genes and miRNA binding sites was performed using: PicTar (version 2006), TargetScan (version 6.0), microRNA.org (August 2010 release), MicroCosm Targets (version 5), DIANA-microT (v3.0 and v4.0), EIMMo [3]. To predict atypical pairing sites, Rna22 algorithm was used [4].

**PLASMIDS**

- pSUPER-pre-miR-210 and pSUPER-scramble plasmids were previously described [5].

- pCDNA3-c-myc-Ago2 is a kind gift of G. Hannon (Cold Spring Harbor Laboratory, New York, USA) [6].

- Plasmids used in luciferase assays were generated by cloning oligonucleotides bearing the wt or deleted miR-210-seed pairing site of ROD1 gene +/- 20 bases, in pMIR-REPORT-Luciferase (pLUC, Ambion) downstream of the stop codon, between SpeI and HindIII restriction sites. A BamHI restriction site was added as control of correct insertion. The following oligonucleotides were used (5’-3’, miR-210 putative binding bases are underlined):

ROD1_forward: CTAGTGGATCCTTGGTCCTCTCACAATCACCTCTTCTGCTGTCACTGGAAGGATGGCCATTCCTGGGGCTAGTA

ROD1_reverse: AGCTTACTAGCCCCAGGAATGGCCATCCTTCCAGTGACAGCAGAAGAGGTGATTGTGAGAGGACCAAGGATCCA

ROD1_del_forward: CTAGTGGATCCTCCTGGAGCTCTTGGTCCTCTCACAATCACCTGGCCATTCCTGGGGCTAGTGGTATACCAGGA

ROD1_del_reverse: AGCTTCCTGGTATACCACTAGCCCCAGGAATGGCCAGGTGATTGTGAGAGGACCAAGAGCTCCAGGAGGATCCA

- As an alternative to chemically modified antisense oligonucleotides, we developed a miR-210 lentiviral decoy “sponge” according to a previous report [7]. Five tandem repeats either of a miR-210 complementary sequence or of a scramble sequence, were cloned downstream of the stop codon of firefly luciferase (derived from pLUC, Ambion, see previous section for details). To prevent RNA interference-type cleavage and degradation of the sponge RNA, a bulge at positions 9-12 was inserted in the miR-210 complementary sequence. The following oligonucleotides were used (5’-3’):

Sponge-210_forward: CTAGTAAAATCAGCCGCTGGTGACGCACAGAAAATCAGCCGCTGGTGACGCACAGAAAATCAGCCGCTGGTGACGCACAGAAAATCAGCCGCTGGTGACGCACAGAAAATCAGCCGCTGGTGACGCACAGAAAAGTCGACA

Sponge-210_reverse: AGCTTGTCGACTTTTCTGTGCGTCACCAGCGGCTGATTTTCTGTGCGTCACCAGCGGCTGATTTTCTGTGCGTCACCAGCGGCTGATTTTCTGTGCGTCACCAGCGGCTGATTTTCTGTGCGTCACCAGCGGCTGATTTTA

Sponge-scramble_forward: CTAGTGTGTAACACGTCTATACGCCCAGTGTAACACGTCTATACGCCCAGTGTAACACGTCTATACGCCCAGTGTAACACGTCTATACGCCCAGTGTAACACGTCTATACGCCCAGTGTAACACGTCTATACGCCCAGTCGACA

Sponge-scramble_reverse: AGCTTGTCGACTGGGCGTATAGACGTGTTACACTGGGCGTATAGACGTGTTACACTGGGCGTATAGACGTGTTACACTGGGCGTATAGACGTGTTACACTGGGCGTATAGACGTGTTACACTGGGCGTATAGACGTGTTACACA

The luciferase-sponge constructs were then sub-cloned in pCCLsin.PPT.hPGK lentiviral plasmid between BamHI and SalI restriction sites.

**SUPPLEMENTARY REFERENCES**

1. Magenta A, Cencioni C, Fasanaro P, Zaccagnini G, Greco S et al. (2011) miR-200c is upregulated by oxidative stress and induces endothelial cell apoptosis and senescence via ZEB1 inhibition. Cell Death Differ 18**:** 1628-1639.

2. Livak KJ, Schmittgen TD (2001) Analysis of relative gene expression data using real-time quantitative PCR and the 2(-Delta Delta C(T)) Method. Methods 25**:** 402-408.

3. Bartel DP (2009) MicroRNAs: target recognition and regulatory functions. Cell 136**:** 215-233.

4. Miranda KC, Huynh T, Tay Y, Ang YS, Tam WL et al. (2006) A pattern-based method for the identification of MicroRNA binding sites and their corresponding heteroduplexes. Cell 126**:** 1203-1217.

5. Fasanaro P, D'Alessandra Y, Di Stefano V, Melchionna R, Romani S et al. (2008) MicroRNA-210 modulates endothelial cell response to hypoxia and inhibits the receptor tyrosine kinase ligand Ephrin-A3. J Biol Chem 283**:** 15878-15883.

6. Karginov FV, Conaco C, Xuan Z, Schmidt BH, Parker JS et al. (2007) A biochemical approach to identifying microRNA targets. Proc Natl Acad Sci U S A 104**:** 19291-19296.

7. Ebert MS, Neilson JR, Sharp PA (2007) MicroRNA sponges: competitive inhibitors of small RNAs in mammalian cells. Nat Methods 4**:** 721-726.
